# Larger Airway Functions in Poultry and Dairy Confinement Workers: A Cross-Sectional Study

**DOI:** 10.7759/cureus.71045

**Published:** 2024-10-07

**Authors:** Mohanarajan Pavithran, Prabhavathi K, Kalyani Praba P, Gisha Sivan, Rachula Daniel, Kanimozhi Sadasivam, Thamarai Selvi K, Saravanan A

**Affiliations:** 1 Physiology, SRM Medical College Hospital and Research Centre, SRM Institute of Science and Technology, Chengalpettu, IND; 2 Medical Research, SRM Medical College Hospital and Research Centre, SRM Institute of Science and Technology, Chengalpettu, IND; 3 Physiology and Stimulation, SRM Medical College Hospital and Research Centre, SRM Institute of Science and Technology, Chengalpettu, IND

**Keywords:** animal workers, dairy workers, large airway function, peak expiratory flow, poultry workers

## Abstract

Background: The peak expiratory flow (PEF) test is a crucial tool for assessing larger airway functions, particularly in individuals exposed to occupational hazards. This study aims to compare PEF values between animal workers (poultry and dairy farm workers) who are regularly exposed to animal allergens and other respiratory irritants and normal workers who do not have such exposures.

Methods: A cohort of animal workers from poultry and dairy farms were evaluated alongside a control group of normal workers without known respiratory hazards. PEF measurements were taken using a computerized spirometer, and results were analyzed to determine differences in respiratory function between the two groups.

Results: There was a significant difference in PEF values, with animal workers exhibiting lower PEF compared to their control group. Further, poultry farm workers had a significant decrease in PEF when compared to dairy farm workers. This study represents a pioneering effort in the comparative analysis of respiratory health among workers in poultry and dairy farms.

Conclusions: The findings indicate that occupational exposure to allergens and irritants associated with animal environments may have deleterious effects on respiratory health. Further analysis, controlling for confounding variables such as smoking status, age, and baseline health conditions, reinforces the initial observation of compromised respiratory function in animal workers. This study underscores the importance of regular respiratory health monitoring and implementation of protective measures for individuals in occupations with high exposure to respiratory irritants. Enhanced awareness and preventive strategies could mitigate the long-term health impacts on animal workers, ensuring safer working environments and improved overall health outcomes.

## Introduction

Occupational lung diseases, also known as work-related respiratory ailments, are conditions resulting from exposure to harmful substances in the workplace. Diagnosing these conditions is critical as it impacts workers' decisions regarding their employment continuation and eligibility for compensation and benefits [1]. The incidence of these diseases is increasingly concerning, with approximately 2 million deaths annually attributed to work-related exposures, underscoring the respiratory risks associated with occupational settings [2]. The term "occupational disease" encompasses any illness contracted due to risk factors arising from work activities [3]. The specific respiratory hazards faced depend on the pollutants workers are exposed to. Occupational respiratory diseases typically stem from prolonged exposure to irritating or toxic substances, leading to acute or chronic respiratory issues. This poses a significant health risk for workers in industries such as poultry farming and dairy farming [2]. Research indicates that individuals in these sectors often experience heightened respiratory problems and reduced lung function, highlighting the occupational health risks associated with intensive animal production [4].

Poultry and dairy farming, as integral parts of the livestock sector, play a crucial role in resource utilization and meeting global protein demands. Increasing incomes, urbanization, and population growth in developing regions drive global demand for animal products, involving a diverse range of workers, from poultry farmers to processing technicians and barn workers. However, these workers face occupational hazards, particularly exposure to harmful toxins, resulting in a higher prevalence of respiratory issues compared to other agricultural workers [5]. Environments in these industries contain airborne particles such as dust, allergens, endotoxins, microorganisms, chemical pollutants, and organic gases, contributing to both acute and chronic respiratory problems among workers.

Epidemiological studies indicate that prolonged exposure to these conditions contributes to chronic respiratory illnesses among workers in these industries [5]. The health effects vary depending on the quantity and frequency of exposure. Continuous inhalation of organic dust can lead to hypersensitive lung conditions, skin allergies, and acute respiratory symptoms like wheezing and sneezing. Additionally, the composition, size, and density of particulate matter in poultry and dairy dust, which includes feed particles, dander, and gases, can impact worker health through aerosolization and bioaerosol contamination. Ammonia, a common contaminant in these environments, exacerbates respiratory issues and can lead to acute or chronic respiratory diseases [6].

Further, organic dusts consist of particles from plants, animals, and microbes, with endotoxin from gram-negative bacteria being a primary component. Components from gram-positive bacteria (peptidoglycans) and fungi (glucans) are also present. Inhalation of organic dust, particularly from hay, may trigger an immune-allergic and inflammatory reaction, contributing to the development of chronic obstructive pulmonary disease (COPD). Bronchial hyperresponsiveness during exposure to organic particles may serve as an indicator associated with the subsequent onset of COPD [7]. Concerns primarily focus on airway obstruction, making lung function assessment critical for identifying poultry and cattle farmers as a higher-risk group for respiratory symptoms.

The peak expiratory flow rate (PEFR) (L/s) is the maximum flow rate achieved during a forceful exhalation from a position of full inhalation [8]. It is an effort-dependent lung parameter that originates from the large airways within approximately 100-120 ms of the start of forced expiration. PEFR primarily reflects the caliber of the bronchi and larger bronchioles, serving as a key measure of effort-dependent airflow. It is a crucial diagnostic and prognostic tool in lung function studies, used to identify airflow limitations, assess their severity, and monitor variations [9]. However, PEFR does not detect small airway obstruction and assesses mainly larger airway obstruction [10]. It is also a good indicator of bronchial hyperresponsiveness, with bronchoconstriction being a key component in the pathophysiology of asthma [11]. There is a lack of data on PEFR among poultry and dairy industry workers in India, highlighting the need for further research in this area. The present study is an attempt to assess the larger airway functions that are significantly reduced in animal workers, specifically those in poultry and dairy farming, compared to non-animal workers, using PEFR.

## Materials and methods

This is a comparative cross-sectional study conducted among 140 participants aged 18 to 55 years. Ethical approval for the study was obtained from the SRM Medical College Hospital and Research Centre Institutional Ethical Committee under reference number SRMIEC-ST0324-1040, dated 03/04/24. Prior to participation, written consent was obtained from all participants, who then underwent a comprehensive assessment including detailed medical and family history, clinical examination, and various investigations.

Inclusion criteria included poultry workers or dairy workers with more than five years of experience. The control group was selected based on their medical history, which showed no previous cases of respiratory symptoms and no history of exposure to either poultry or dairy farming. Exclusion criteria included workers below 18 and above 55 years, smokers, pregnant women, and previous history of respiratory illness or cardiovascular diseases. The sampling technique adopted was stratified random sampling. The expected prevalence was 27.9 based on which the sample size was calculated.

Among the 140 participants, 70 individuals were animal workers, out of which 35 were involved in poultry farming and 35 in dairy farming, and the control group comprised 70 individuals who were non-animal workers. The subjects were randomly selected from each strata using the lottery method. The poultry workers were exposed to dust, dander, ammonia, poor ventilation, and heat stress, whereas dairy workers were exposed most to organic dust, ammonia, methane, and physical injuries as they were handling larger animals. The study was conducted at SRM Medical College Hospital and Research Centre, Tamil Nadu, focusing on evaluating pulmonary abnormalities from April 2024 to July 2024.

**Figure 1:**
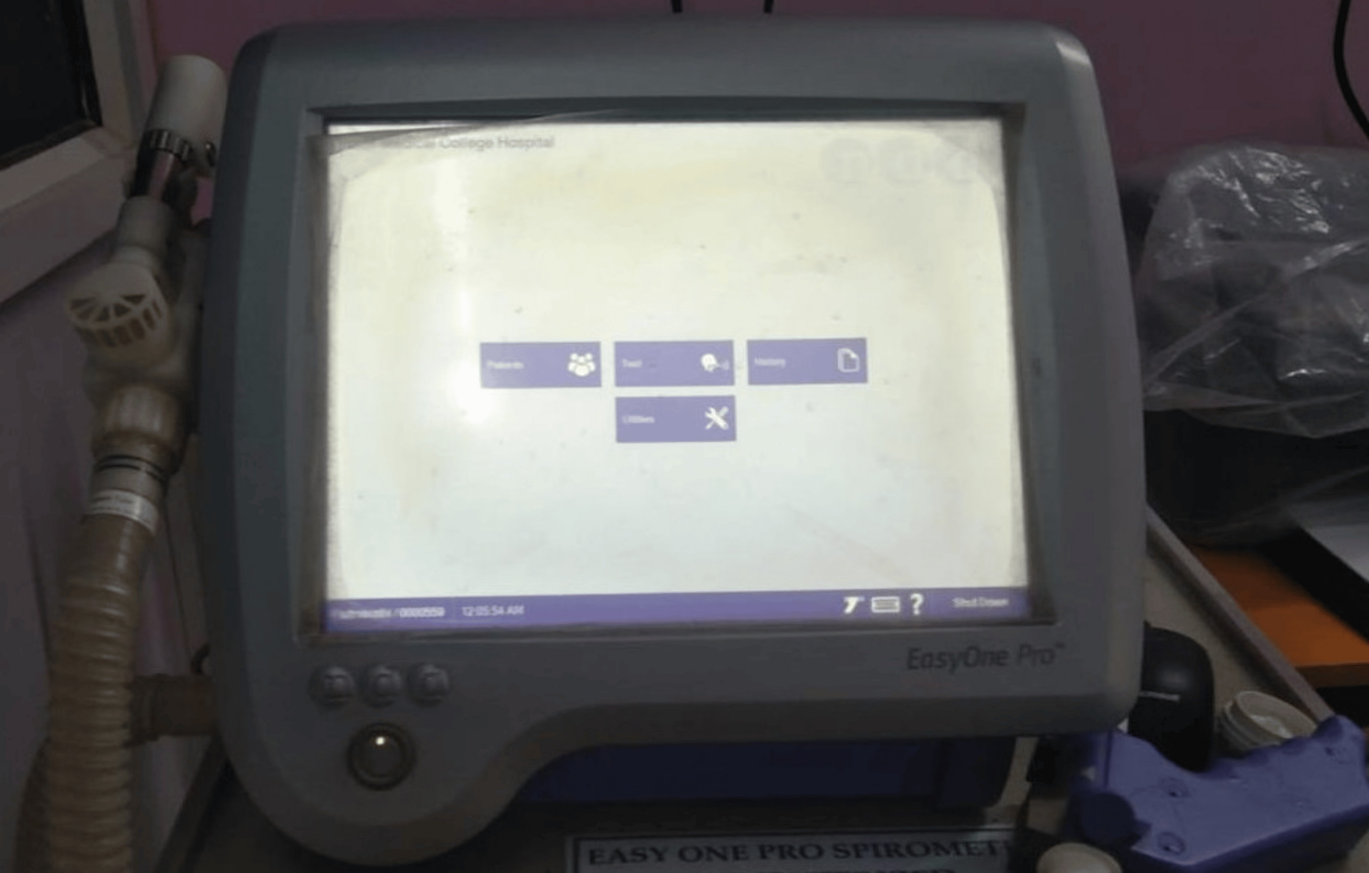
Computerized spirometer used to analyze pulmonary function test

All participants underwent anthropometric measurements, physical examinations, and PEFR tests. PEFR was measured using the EasyOne Pro Computerized Spirometer respiratory analysis system, produced by Medizintechnik AG in Zurich, Switzerland. The patient was explained about the test. The patient was seated with the neck slightly elevated. After attaching a clean mouthpiece, the patient was instructed to take a full deep breath, seal lips tightly around the mouthpiece, and exhale as hard and fast as possible in a single blow until the lungs were empty. This process was repeated at least three times, with sufficient rest between attempts, ensuring no coughing or hesitation during the exhalation. The highest PEFR value from the three acceptable attempts was recorded in liters per second (Figure 1) [12-14].

Statistical analysis

Statistical analysis was performed by a Student's t-test to compare the means of controls and cases (poultry and dairy farm workers), followed by a one-way ANOVA with a significance level of p-value <0.05. The data were statistically analyzed by SPSS Statistics version 20.0 (IBM Corp. Released 2011. IBM SPSS Statistics for Windows, Version 20.0. Armonk, NY: IBM Corp.).

## Results

The mean age of the controls was 26 ± 4.8 years and that of the animal workers was 34.4 ± 8.9 years. The age comparison across the three groups control, poultry workers, and dairy workers was as follows: control group was 26 ± 4.8 years, poultry workers were 34.1 ± 9.69 years, and dairy workers were 34.77 ± 8.25 years (Table 1).

**Table 1:** Anthropometric characteristics of the study population BMI: body mass index

Variables	Control	Poultry workers	Dairy workers
Weight (kg)	60.2 ± 9.68	62.91 ± 9.27	69.08 ± 9.80
Height (cm)	159.6 ± 9.20	156.2 ± 8.32	162.2 ± 10.4
BMI	23.8 ± 4.61	25.86 ± 0.71	26.3 ± 4.12

The gender distribution across the control and animal farm worker groups is as follows: in the control group, males constitute 64.2% and females 35.8%. Among the animal farm worker group, males represent 41.4% and females 58.6%. Further, the gender distribution among the poultry and dairy worker groups is as follows: in the poultry workers, 37.2% are males and 62.8% are females, and in the dairy workers, males account for 45.8% and females for 54.2%. The distribution of BMI among the study groups is as follows: in the control group, 61.4% are normal weight, 25.7% are overweight, and 12.8% are obese. Among poultry workers, 37.1% are normal weight, 48.5% are overweight, and 14.2% are obese. Dairy workers have 34.2% in the normal BMI range, 54.2% overweight, and 11.4% obese (Table 1).

**Figure 2:**
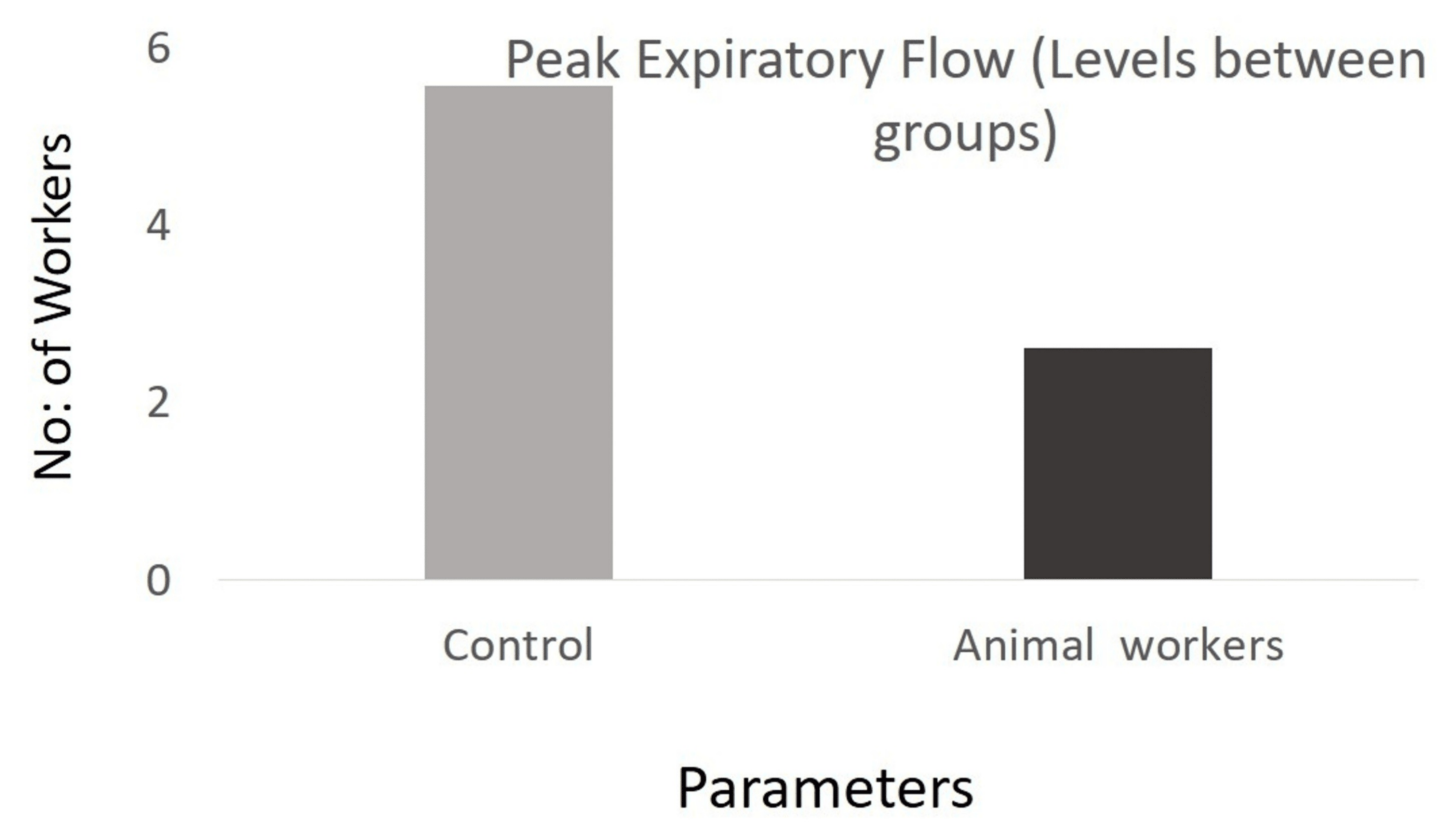
Comparison of PEF (L/s) mean levels between groups PEF: peak expiratory flow

**Figure 3:**
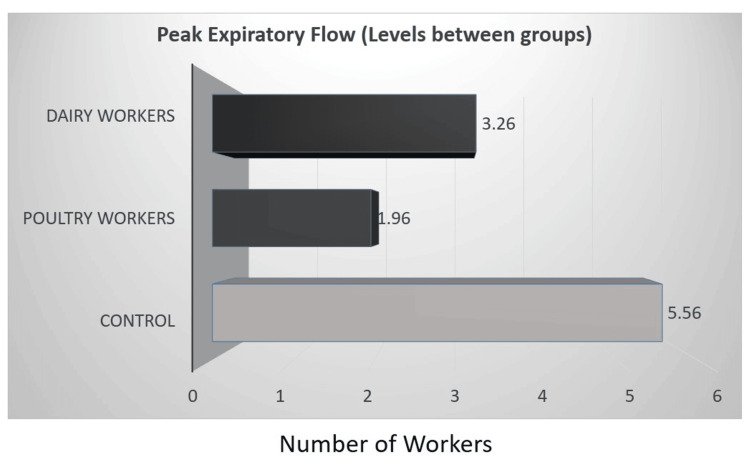
Comparison of PEF (L/s) mean levels between groups PEF: peak expiratory flow

A comparison of peak expiratory flow (PEF) between the control group and animal farm workers using an independent sample t-test was notably higher at 5.56 L/s (SD = 1.95, SE = 0.23) compared to animal farm workers, who had a mean PEF of 2.61 L/s (SD = 1.70, SE = 0.20). The t-test results demonstrated a statistically significant difference in PEF between the groups, with a p-value of less than 0.0001 (Figure 2). Further, the ANOVA results showed a significant difference in PEF among groups with a p-value <0.0001. The control group had a notably higher mean PEF of 5.56 L/s (SD = 1.95, SE = 0.23), compared to poultry workers and dairy workers, whose mean PEF values were 1.96 L/s (SD = 0.81, SE = 0.13) and 3.26 L/s (SD = 2.08, SE = 0.35), respectively (Figure 3).

## Discussion

Animal workers, due to their exposure to poultry and dairy dust at their workplaces, experience higher rates of illness and mortality from respiratory diseases than anticipated. Despite this being recognized for centuries, it continues to be a significant issue [15]. It is well documented that these workers are susceptible to various work-related diseases affecting the cardiovascular and respiratory systems [16]. Respiratory diseases notably contribute to occupational disabilities, with immune mechanisms likely playing a crucial role in many of these conditions. Respiratory problems in animal workers arise from dust exposure during activities such as surface brushing and litter spreading, worsening conditions like asthma and chronic bronchitis. Protecting the health of these workers is essential.

Pulmonary function abnormalities are prevalent among animal farmers, aligning with findings from previous research. The study reveals significant differences in age distribution among control and animal farm workers. The animal workers involved in animal farming are more likely older individuals, indicating a shift toward older individuals in animal farming, which goes along with the National Agricultural Workers Survey tables for 2013-2014. Knowing these demographics helps tailor interventions for age-specific health issues and implement preventive measures for the well-being of poultry and dairy workers.

The gender distribution among poultry and dairy cattle workers shows a greater number of females, especially in dairy farming, underscoring the vulnerability of women in these sectors. Thus, targeted interventions and workplace health initiatives are crucial to meet the specific needs and vulnerabilities of female dairy and cattle workers. A study highlighted increased female participation in dairy farming [3]. Further, dairy workers had a higher percentage of overweight compared to poultry workers, potentially due to the physical demands and dietary habits associated with their work.

A comparison of PEF between the control group and animal farm workers suggests that both poultry and dairy workers experienced a substantial reduction in their ability to exhale forcefully, with poultry workers being the most affected. Further, PEF was comparatively less in poultry workers when compared to dairy workers. The mechanism behind the decrease in PEFR would be likely due to the enlargement of mucosal cells due to irritation from poultry and dairy dust, leading to increased mucus secretion and the formation of mucosal plugs that obstruct exhaled air [17]. The dust particles accumulate in the airways, exacerbating these effects, which are more observed in poultry farmers [18]. Various studies have documented reduced lung function among workers in the swine and poultry industries [19-20]. Occupational asthma is frequently observed among poultry workers [21]. Swine barn workers similarly experience lower PEFR due to heightened airway reactivity [22]. Even Dutch veterinarians show variable PEFR values due to their exposure to dust [23]. Components of poultry dust, including endotoxin, mites, grain particles, and wood dust, contribute to airway inflammation and increased airway sensitivity [24]. These factors lead to inflammatory changes in the lung epithelium [25].

A study observed that inhaling organic dust can trigger an acute inflammatory response in the airways and cause fever in individuals who are not sensitized, a condition known as toxic pneumonitis or organic dust syndrome [26]. Furthermore, exposure to high levels of dust can lead to increased phlegm production and pulmonary inflammation within four to 10 hours after exposure, with symptoms potentially lasting up to 24 hours. Chronic exposure to such dust, on the other hand, may result in bronchitis and asthma [27]. The study benefits from recording PEFR in poultry and dairy workers. A similar study has shown that poultry workers exposed to higher concentrations of dust have lower airway function when compared to other workers [28]. Further, poultry farms have higher concentrations of dust and particulate matter, endotoxins, and ammonia compared to dairy farms. The microbial load, including bacteria, molds, and fungi, is often higher in poultry environments. Poultry workers often involve close contact with birds and their waste, leading to more direct exposure to respiratory irritants, while dairy farm workers, while still involving exposure to organic dust and gases, generally involve larger and more open spaces, which can help disperse harmful substances more effectively. Also, poultry workers are more in confined and enclosed spaces compared to dairy farm workers, which could be the reason for more declining lung functions. Due to these factors, poultry farm workers are at a higher risk of developing respiratory issues and experiencing greater declines in lung function compared to their counterparts in dairy farming [5]. However, a more comprehensive understanding could be achieved by correlating dust levels, control measures in housing, and conducting environmental assessments of poultry and dairy confinement buildings.

A lower than-normal PEFR indicates that the larger airways may be narrowed or obstructed. Regular PEFR testing can thus help identify patients at risk of severe asthma attacks or exacerbations, enabling preventive measures to be implemented. Patients can use PEFR to identify potential triggers that worsen their symptoms. By recording this alongside the symptom diaries, individuals will be able to link decreases in lung function to specific environmental-related triggers. The test is simple and can be performed easily, encouraging patients to take an active role in managing their respiratory health. It fosters awareness of their condition and promotes adherence to treatment plans. Thus, the PEFR test is an easy and valuable tool for assessing and monitoring lung function, guiding treatment, and improving patient outcomes in respiratory diseases.

Public health and policy implications for poultry and dairy farm workers experiencing decreased PEFR are significant, as these individuals are often exposed to various respiratory hazards, including organic dust, allergens, and harmful gases. Effective policies should focus on enhancing workplace safety standards by implementing proper ventilation systems, providing personal protective equipment, and promoting regular health screenings for respiratory function among workers. Education and training programs that inform workers about the risks associated with their occupations and the importance of monitoring their lung health can empower them to take proactive measures [29]. Furthermore, policies should advocate for research into the long-term health effects of occupational exposure in these industries, guiding interventions to mitigate risks. Collaborating with public health agencies to establish guidelines for safe working conditions, as well as supporting initiatives that promote respiratory health, can significantly reduce the incidence of respiratory diseases among poultry and dairy workers. Such measures not only protect the health of these workers but also contribute to a healthier workforce and enhanced productivity in the agricultural sector [30].

Limitations

This study on respiratory health among animal workers presents several limitations. Firstly, gender distribution also reveals a higher proportion of females in dairy farming, yet the study lacks specific analyses on how gender-related physiological differences might impact respiratory outcomes. Furthermore, the study's cross-sectional design limits the ability to establish causation between dust exposure and respiratory conditions, and the absence of long-term follow-up prevents an understanding of chronic effects. Comprehensive environmental assessments and more detailed exposure measurements are needed to fully elucidate the relationship between dust exposure and respiratory health in these occupational settings. Also, adequate statistical analysis has to be done to overcome the confounding factors with respect to age, gender, and BMI.

## Conclusions

The PEFR test plays a crucial role in assessing the larger airway functions of poultry and dairy workers. By measuring PEFR, potential respiratory issues caused by exposure to dust and other pollutants in these occupational environments can be detected early. The decreased PEFR among poultry workers, compared to dairy farm workers and non-farm workers, highlights a significant occupational health challenge that demands urgent attention. Poultry workers face elevated exposure to organic dust, allergens, and harmful gases during handling and processing, leading to a higher prevalence of respiratory issues that adversely affect their quality of life and productivity. In contrast, while dairy workers also encounter respiratory risks, their exposure is typically less concentrated. To address these health implications, robust workplace safety policies are essential, including improved ventilation, personal protective equipment, and regular health monitoring. Additionally, education and training programs to raise awareness about respiratory risks and PEFR monitoring can empower workers to take proactive health measures. By fostering collaboration between public health agencies, agricultural employers, and workers, targeted interventions can mitigate respiratory illness risks, promoting a healthier workforce and enhancing the overall sustainability of the agricultural sector.
